# Effects of estrogen on spatial navigation and memory

**DOI:** 10.1007/s00213-024-06539-3

**Published:** 2024-02-26

**Authors:** Gina Joue, Tobias Navarro-Schröder, Johannes Achtzehn, Scott Moffat, Nora Hennies, Johannes Fuß, Christian Döller, Thomas Wolbers, Tobias Sommer

**Affiliations:** 1 Institute of Systems Neuroscience, University Medical Center Hamburg-Eppendorf, Martinistraße 52, 20246 Hamburg, Germany; 2 Kavli Institute for Systems Neuroscience, Norwegian University of Science and Technology, Olav Kyrres Gate 9, 7030 Trondheim, Norway; 3 Department of Neurology with Experimental Neurology (CVK), Charité Universitätsmedizin Berlin, Augustenburger Platz 1, 13353 Berlin, Germany; 4 School of Psychology, Georgia Institute of Technology, 654 Cherry Street, Atlanta, GA 30332 USA; 5 Institute of Forensic Psychiatry and Sex Research, University Duisburg-Essen, Hohlweg 26, 45147 Essen, Germany; 6 Max Planck Institute for Human Cognitive and Brain Sciences, Stephanstraße 1a, 04103 Leipzig, Germany; 7 German Center for Neurodegenerative Diseases (DZNE), Leipziger Straße 44, 39120 Magdeburg, Germany

**Keywords:** Spatial navigation, Spatial memory, Estrogen, Sex differences, Gender differences

## Abstract

**Rationale:**

Animal studies suggest that the so-called “female” hormone estrogen enhances spatial navigation and memory. This contradicts the observation that males generally out-perform females in spatial navigation and tasks involving spatial memory. A closer look at the vast number of studies actually reveals that performance differences are not so clear.

**Objectives:**

To help clarify the unclear performance differences between men and women and the role of estrogen, we attempted to isolate organizational from activational effects of estrogen on spatial navigation and memory.

**Methods:**

In a double-blind, placebo-controlled study, we tested the effects of orally administered estradiol valerate (E2V) in healthy, young women in their low-hormone menstrual cycle phase, compared to healthy, young men. Participants performed several first-person, environmentally rich, 3-D computer games inspired by spatial navigation and memory paradigms in animal research.

**Results:**

We found navigation behavior suggesting that sex effects dominated any E2 effects with men performing better with allocentric strategies and women with egocentric strategies. Increased E2 levels did not lead to general improvements in spatial ability in either sex but to behavioral changes reflecting navigation flexibility.

**Conclusion:**

Estrogen-driven differences in spatial cognition might be better characterized on a spectrum of navigation flexibility rather than by categorical performance measures or skills.

**Supplementary Information:**

The online version contains supplementary material available at 10.1007/s00213-024-06539-3.

## Introduction

Although males (M) and females (F) are generally believed to differ in spatial navigation abilities, with males having the advantage (Hornung et al. 2020; Nazareth et al. 2019; Williams and Meck 1991), extensive animal and human studies cannot offer conclusive accounts (Clint et al. 2012; Coluccia and Louse 2004; Nazareth et al. 2019). Among purported differences is men generally perform better on hippocampal-dependent tasks as can be tested by mazes (de Castell et al. 2019; Harris et al. 2019; Moffat et al. 1998) or wayfinding (Montello et al. 1999), where a *place* or *allocentric* navigation strategy is more efficient (Sandstrom et al. 1998; Saucier et al. 2002). This strategy involves establishing spatial relationships between environment cues (Biegler and Morris 1993) and generalizing them into flexible *cognitive maps* (Tolman 1948; Downs and Stea 1973). Women, in contrast, generally adopt a *response* or *egocentric* strategy, locating landmarks relative to themselves, or using internal cues (Tolman 1948), and formulate routes in terms of procedures (Andersen et al. 2012; de Castell et al. 2019; Sandstrom et al. 1998). They commit more errors and are slower in tasks where an allocentric strategy would be advantageous, for example, pointing towards locations seen along a route (*path integration*; Bell and Saucier 2004; Ishikawa and Montello 2006), or following directions with Euclidean distances and cardinal points (Saucier et al. 2002). Moreover, women’s spatial navigation strategies vary with the menstrual cycle (Brown et al. 2023). However, differences between the sexes are not consistently reported (Levy et al. 2005). Moreover, both sexes, in animal and human studies, successfully alternate strategies when required (Astur et al. 2016; Ferguson et al. 2019; van Gerven et al. 2016; Williams and Meck 1991), leading to proposals that strategy bias describes differences better than ability (Andreano and Cahill 2009; Boone et al. 2018; Korol and Pisani 2015).

Despite the contention over sex-based differences in spatial navigation, hormone-dependent physiological changes that sometimes lead to altered spatial performance provide a compelling, mechanistic reason for such differences (Duarte-Guterman et al. 2015; Frick et al. 2018). The differences in hormone concentrations between males and females during development result in *organizational* effects, that is, permanent structural or cellular differences (Williams and Meck 1991, 1993). Fluctuations in circulating hormone concentrations after development have *activational* effects (Holden and Hampson 2021), which in contrast to organizational effects are reversible (Arnold and Breedlove 1985). The female sex hormone 17-$\beta$ estradiol (E2) plays a key role in both organizational and activational differences between the sexes in regulating physiology and behavior (Galea et al. 2017; Wu et al. 2009), as do estrogen receptors (e.g., ER$\alpha$ action on inhibitory neurons: Wu and Tollkuhn 2017). Although often considered a female sex hormone, estrogen is also an important neuromodulator in males, as reflected by the non-negligible density of E2 receptors in the male brain (Frick et al. 2015). Moreover, the “male” sex hormone testosterone (TST) is aromatized to E2, which is argued to account for some of the behavior changes associated with TST (Williams and Meck 1993; Wu et al. 2009; however androgen metabolites and signaling beyond E2 are also important: MacLusky et al. 2006; Sato et al. 2004). Aromatization (i.e., de novo synthesis in both sexes) notably occurs in the neurons in the hippocampus (Hc) (MacLusky et al. 2006; Hojo et al. 2008) and the prefrontal cortex (Fantie and Kolb 1990), brain areas consistently implicated in spatial navigation (Burgess et al. 2002; Negrón-Oyarzo et al. 2018). In fact, E2 acts as a neuromodulator mainly in these two areas (Luine and Frankfurt 2020).

The long-term, organizational effects of E2 exposure are supported by developmental animal studies, which unlike human studies — barring rare genetic diseases such as prenatal exposure to excess androgen in females with Congenital Adrenal Hyperplasia (CAH) or androgen insensitivity syndrome (AIS) where a genetic mutation results in partial or complete absence of androgen receptor activity and hence effectively no exposure to TST — allow us to separate organizational hormone effects from activational effects. Animal studies and models provide us the opportunity to directly investigate the mechanistic and physiological reasons that might be driving any differences between the sexes.^1^

E2 also has activational effects related to spatial navigation cognition. Circulating endogenous (Woolley and McEwen 1992) and acute, exogenous (Gould et al. 1990) elevations of E2 can both increase dendritic spine density and neurogenesis, as well as, facilitate long-term potentiation (Ooishi et al. 2012). Such morphological changes when observed in the hippocampus are associated with improved learning in spatial navigation tasks (Kitabatake et al 2007; Isgor and Sengelaub 1998; Velázquez-Zamora et al. 2012). E2 neuromodulation occurs either as rapid effects — i.e., within hours via membrane-bound estrogen receptor $\alpha$ and $\beta$, or G protein-coupled estrogen receptors (ER$\alpha$, ER$\beta$ and GPER, respectively) — or slower “classical”, genomic effects that take place after about 24 hours via cytosolic ER$\alpha$/$\beta$ receptors (Fx-r.SD: Graves et al. 2011). Both rapid and genomic effects are associated with enhanced spatial navigation performance in both sexes (Daniel et al. 1997; Duarte-Guterman et al. 2015; Luine et al. 1998; Phan et al. 2011). They provide a physiological explanation for why intact female rats have been found to perform better on allocentric strategy-based tasks (strategy-switching variant of the Y-maze, r.SD: Korol and Kolo 2002; MWM, r.LE: Warren and Juraska 1997; Korol et al. 2004) during the pre-estrous phase, when E2 levels are naturally high and hippocampal cells have greater dendritic number and density than during the low-E2 and lower dendritic density estrous phase (r.SD: Woolley and McEwen 1992; González-Burgos et al. 2005). Conversely, ovariectomized rats not administered E2V can perform better on an egocentric spatial navigation task (x-r.SD: Korol and Kolo 2002; r.LE: Davis et al. 2005).

However, some animal (rodents in an MWM: Berry et al. 1997) and human studies with naturally cycling women (RAM, RAM and path integration task, respectively:Hussain et al. 2016; Patel et al. 2022; Brown et al. 2023) have reported no performance differences when females were in their naturally low versus high E2 phases. In more challenging navigation tasks requiring allocentric strategy such as the MWM, reported differences between men and women or E2-modulated differences in overall performance success or errors are also mixed (e.g., compare Astur et al. 2004; de Castell et al. 2019). On the other hand, these more difficult tasks support the use of richer and more illustrative measures of spatial cognition, like navigation patterns (e.g., speed or spatial coverage) and path integration, which have been argued to better capture any hormone-concentration dependent differences (Nazareth et al. 2019; Piber et al. 2018; Patel et al. 2022). In general, E2 effects are subtle and sensitive to factors such as task type and difficulty, with effects increasing with difficulty (Jonasson 2005; Voyer et al. 1995) and more real-life like environments as opposed to laboratory mazes (Montello et al. 1999; Saucier et al. 2002; Tlauka et al. 2005).

Although E2 modulates physiological changes in the neurons in the hippocampus in male rats like in females (Frick et al. 2018) and can also improve allocentric performance, the magnitude of the effects differ between males and females. Some studies have reported greater E2 effects (exogenously elevated) in males (e.g., r.SD in RAM with longer delays: Luine and Rodriguez 1994). Such behavioral differences have been attributed to organizational differences in the brain such as in E2 signaling, distribution of specific ERs (reviews: Frick et al. 2018; Gillies and McArthur 2010; r.SD: Jain et al. 2019), and also highlight the interaction with other hormones like TST and progesterone (reviews: Le et al. 2020; Pletzer et al. 2019.

The complicated interplay of all these factors contribute to the difficulty in being able to generalize and draw clear conclusions from group-averaged effects (Frick et al. 2015). Our goal in this study was to characterize differences between the sexes in navigation behavior and strategy more comprehensively in a carefully controlled placebo-controlled study, first by disentangling the effect of circulating E2 from differences between the sexes when E2 levels are comparable. We therefore orally administered estradiol valerate (E2V) to healthy young men, and women in their low-hormone follicular phase. E2 levels in the male and female placebo groups were therefore equally low. Participants in the test group took E2V over two consecutive days before performing tasks to allow both rapid and genomic effects of E2 to take place. Keeping in mind that any differences might be nuanced and sensitive to task choice and ecological validity (Tlauka et al. 2005), we adopted three 3-D spatial navigation tasks to approximate the complexities of real-world navigation within a laboratory setting. Two were inspired by animal paradigms (Y-maze and the MWM) commonly used to test the effects of hormones on learning in animal studies (Dohanich 2002) and to tap into different aspects of spatial cognition (Astur et al. 2004). Having several tasks allowed us to better characterize the finer potential enhancing effects that E2 might have on navigation strategy and behavior (Dohanich 2002; Korol et al. 2004; Duarte-Guterman et al. 2015).

## Materials and methods

### Participants

The data reported here are from 66 young adult men (mean age of 26.0 ± SD 3.7 yrs) and 63 naturally cycling young women in their low-hormone early follicular phase (age 26.1 ± SD 3.9 yrs). Participants were randomly assigned, double-blind, to receive estradiol valerate (E2V) or placebo (PBO) pills, resulting in four groups: 30 females on PBO (F.PBO; age 26.4 ± 3.7 yrs, BMI 22.4 ± 2.6, tested 0.5 ± 3.1 days after menses onset), 33 females on E2V (F.E2V; age 25.9 ± 4.1 yrs, BMI 21.8 ± 2.4, tested 1.85 ± 3.1 days after menses onset), 32 males on PBO (M.PBO; age 26.1 ± 3.6 yrs, BMI 23.4 ± 2.3) and 34 men on E2V (M.E2V; age 26.0 ± 3.6 yrs, BMI 23.2 ± 2.9).

One of the three tasks (arena) was conducted in an MRI scanner (neuroimaging data reported elsewhere). Seven participants did not complete the arena task due to claustrophobia (2 men and 4 women) or scanner equipment problems (1 man) and were therefore excluded from all analyses. One woman was excluded for high E2 and progesterone (P4) concentrations that were more typical of mid-luteal phase.

Participants were recruited through the University of Hamburg website. All participants were right-handed and reported to be free of psychiatric illnesses, to not be users of illicit drugs or central nervous medication, and not be regular smokers. None of the participants had contraindications for taking E2V (e.g., obesity or at risk for cardiovascular problems) or for MR examinations. Only naturally cycling women who had not taken any oral contraceptives or were not pregnant in the 6 months prior to the study were included. Menstrual cycle timing was determined by self reports and forward counting, then verified by saliva hormone concentrations. Ethics approval was obtained from the Ethics Committee of the Hamburg Medical Association (PV4738). All volunteers gave written, informed consent for this study and received monetary reimbursement.

### Hormone assessment

Estradiol valerate (E2V) is the synthetic esther of natural estrogen, with an average tmax of approximately 3 to 6 hours and a half-life of 14 hours (Kuhl 2005). For the E2V groups, women received 8 mg and men 12 mg E2V per day over two days. This dosage, based on our previous study (Bayer et al. 2018) and a pilot study, was chosen to bring the E2 levels of men and women to within the same range, at the high end of the natural physiological range in women.

**Fig. 1:**
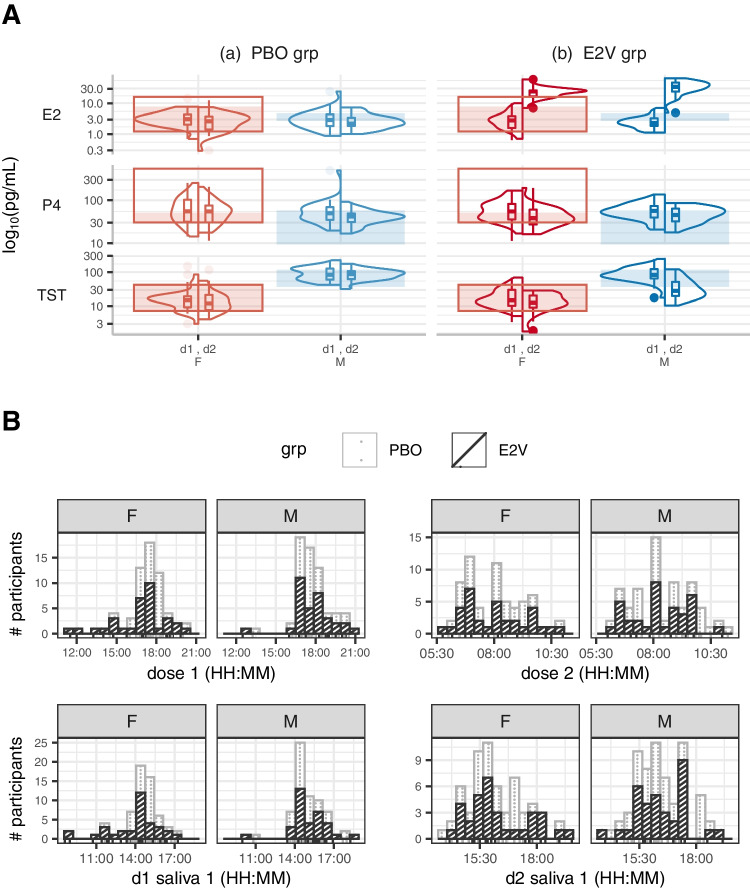
Hormone levels with times of testing across groups. **A (top)** Distributions of hormone levels measured in saliva of 17$\beta$-estradiol (E2), progesterone (P4) and testosterone (TST) are shown for men (M) and women (F) in the placebo- (PBO) and estradiol-administered (E2V) groups prior to administration on day 1 (d1) and after treatment on day 2 (d2). Shaded areas are the expected concentrations for the age range (and follicular phase for the women) of our participants, as published by the manufacturer of the hormone assays used. The box outlines delimit the expected physiological range across the entire menstrual cycle for the age range of our female participants. Daily E2V administration of 8 mg for women and 12 mg for men over two consecutive days increased E2 concentrations in both men and women and decreased TST concentrations in men. **B (bottom)** Stacked bar charts and rug charts, by treatment group, showing number of participants cumulative across groups (# participants) who received doses and had saliva collected at the time specified (HH:mm, 24-h clock). To avoid diurnal fluctuations in hormone levels, participants were tested in the afternoon as close to the same times as was practical. They received the first dose (dose 1) of E2V (dark, cross-hatched bars) or PBO (light, dotted bars) capsules in the afternoon on day 1 and took the second dose (dose 2) the following morning. Hormone levels were measured from saliva samples collected several times over an hour to ensure stable assessment. Shown here are the times on a 24-h clock of the first sample collected on each day. On day 1 (d1), saliva was sampled before the first dose to test baseline hormone concentrations. On day 2 (d2), about 8 hours following the second dose, i.e., late afternoon, participants completed the Y-maze, arena, and town tasks. The first task was started about an hour before the first saliva sample was collected (the times shown in the bottom right panel)

On Day 1 of the experiment, the first dose of E2V or PBO (visually identical capsules) was administered double-blind by the experimenter in the afternoon. Participants took the second dose on their own the next morning, approximately 6 h before the start of the first task (median start time around 16:45) when E2 levels were expected to peak (Fig. 1A for dose and task start/saliva collection times).

On each testing day, three saliva samples were collected over about an hour and pooled for analysis ($\sim$3 mL in total) in order to achieve stable hormone level assessments. Time lapse between second dose and when first saliva sample was collected was about 8.1±0.76 h. Although blood was drawn ($\sim$1 mL) on Days 1 and 2 from participants who consented, they were primarily assessed for the E2V groups. Therefore, only hormone concentrations in saliva are reported here. Saliva samples were stored at −18^∘^C until analysis by IBL (Hamburg, DE) using highly sensitive luminescence assays for salivary E2, P4 and TST.

### Tasks

**Fig. 2:**
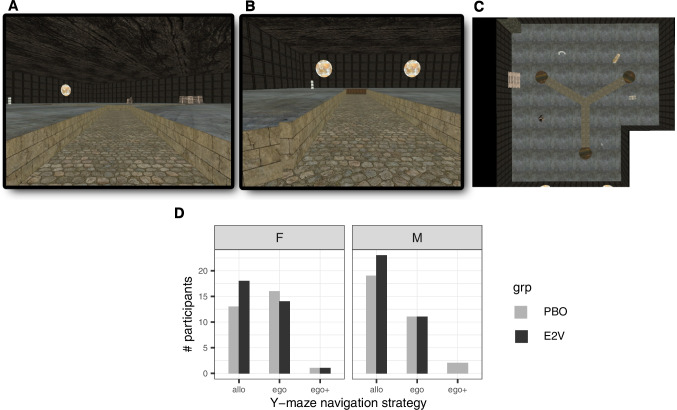
Y-Maze **A (top left)** Player view from start of training block. **B (top middle)** Player view from maze center hub of target arm (flanked by yellow discs as extramaze cues). **C (top right)** Aerial view. **D (bottom)** Number of participants who adopted an allocentric (allo) or egocentric (ego) strategy in each of the four groups: women (F) or men (M) administered placebo (PBO) or estradiol (E2V). Participants who navigated egocentrically but noticed a change in extramaze cues (ego$+$) were excluded from analyses

**Fig. 3:**
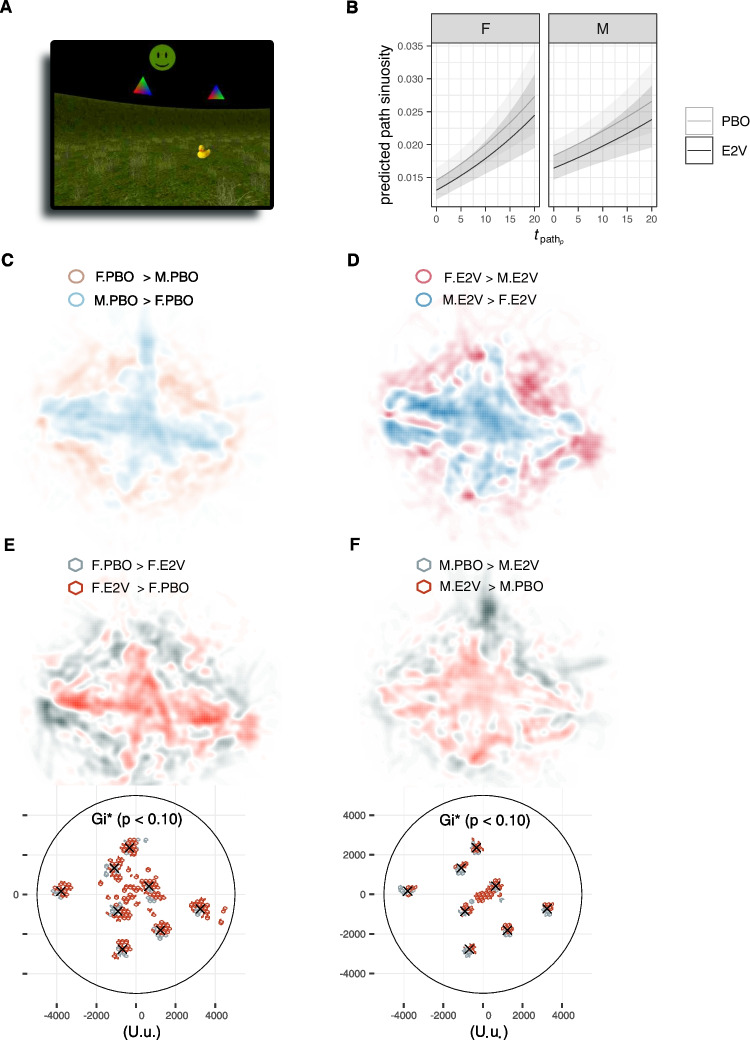
Spatial biases during navigation in the arena. Players moved in first-person viewpoint in the arena with a radius of 5000 Unreal units (U.u.), equivalent to 16 virtual meters. The only cues were the banks of the arena and distal rainbow-colored triangles on the horizon. **A** A feedback scene is shown where the player had just dropped off an object (rubber duck) relatively close to its correct location (green and happy smiley) and the object has reappeared in the arena to be collected. **B** Movements such as path sinuosity differed between groups and over time. Shaded areas show confidence intervals of predictions based on standard errors. **C**–**F** Several difference heat maps are shown comparing how groups moved through the arena. The top row of heat maps, **C** and **D**, compare the sexes (men/M in cold colors, women/F in warm colors) on placebo (PBO) and estradiol valerate (E2V), respectively. Heat maps **E** and **F** compare E2V (dark red) and PBO (gray) for women and men, respectively, and are shown with the corresponding local Getis-Ord $G_{i}^*$ statistical maps thresholded at $p<0.10$. Both E2V groups spent more time around the central areas of the arena than PBO groups. The black crosses on the $G_{i}^*$ statistical maps mark the eight target object locations

**Fig. 4:**
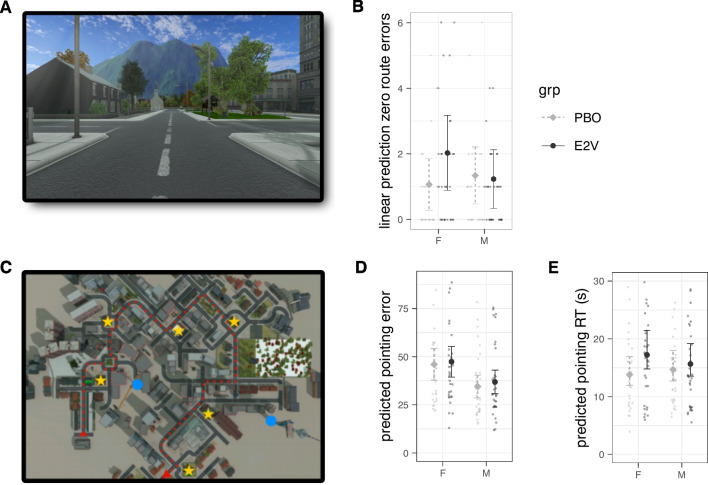
Town task layout and model predictions on retracing route errors, landmark orientation pointing accuracy and RT. **A** A scene from the player’s viewpoint while learning the route through the town. **B** Retracing the route with no errors was more than twice as likely in women (F) on placebo (PBO) than on estradiol valerate (E2V). No treatment differences were found in men (M). **C** Aerial view showing the route learned (dotted red line) and the landmarks (yellow stars) participants were tested in the surprise landmark location pointing test. Blue dots mark the locations of tall landmarks that were always visible to facilitate allocentric referencing. **D** Elevated E2 did not affect pointing errors, but sex reliably predicted pointing errors, which are higher in women. **E** Neither sex nor treatment predicted pointing RT, but women on E2V were slightly slower than women on PBO. Error bars are standard error of the mean. Actual data points are also shown

Three previously developed 3-D computer simulations of spatial navigation tasks were used. In order of increasing task complexity, these were (1) a Y-maze, effectively a radial arm maze with three arms and a variant of the T-maze, to probe whether initial navigation strategy bias during wayfinding was allocentric or egocentric (Parizkova et al. 2018; Rodgers et al. 2012; Fig. 2A–C for player’s viewpoint and bird’s eye view of the environment); (2) a circular arena, inspired by the MWM task (Morris 1981), with no intramaze cues so navigation must rely on orientation with respect to extramaze cues and distance estimations, thus testing allocentric navigation strategy (Navarro Schröder et al. 2015; Fig. 3A for player’s viewpoint); and (3) a town to investigate route learning in a real-world-like landmark-rich environment (Craig et al. 2019) and the development of cognitive maps when wayfinding and exploring an environment in an episodic egocentric fashion (transition to hippocampal-dependent navigation and memory; Fig. 4A and B for a scene from player’s viewpoint and bird’s eye view of the environment). The first two tasks were inspired by paradigms commonly used in animal studies to test the effects of hormones on learning and memory (Dohanich 2002). To reinforce ecological validity, all games were played in first-person viewpoint.

### Y-maze

The Y-maze, developed on the Unreal Tournament 2003 gaming engine (Epic Games, Rockville, MD, USA) and used in previous studies (Parizkova et al. 2018; Rodgers et al. 2012), helped determine participants’ bias towards an allocentric or egocentric navigation strategy. The Y-maze is an indoor “maze” of 3 straight arms of equal length, radiating at equal angles from each other (i.e., angle between adjacent pairs of arms was 120^∘^; Fig. 2C). The maze was sunken at partial player height in a closed room where room decorations outside of the maze served as landmarks. Participants used a keyboard to move within the maze in first-person viewpoint and were instructed to find the corridor (arm of the maze), at the end of which they heard a harmonious tone, as often as the game repeated. Reaching the end of the wrong corridor triggered a discordant buzzer. As soon as participants reached the end of either corridor and triggered a sound, they were transported back to the same starting arm and had to reach the target location again.

Participants were not informed that the task was divided into a training block with a fixed starting point and finished with a probe trial where they were placed in a different arm of the maze to probe whether they would navigate allocentrically or egocentrically. That is, the end of one arm was the fixed starting point during the training block (Fig. 2A player’s viewpoint at the start of each training trial), a second arm the target arm with a harmonious tone, and the remaining third arm the wrong/dissonant arm during the training block but also the starting point during the probe trial (Fig. 2B).

**Fig. 5:**
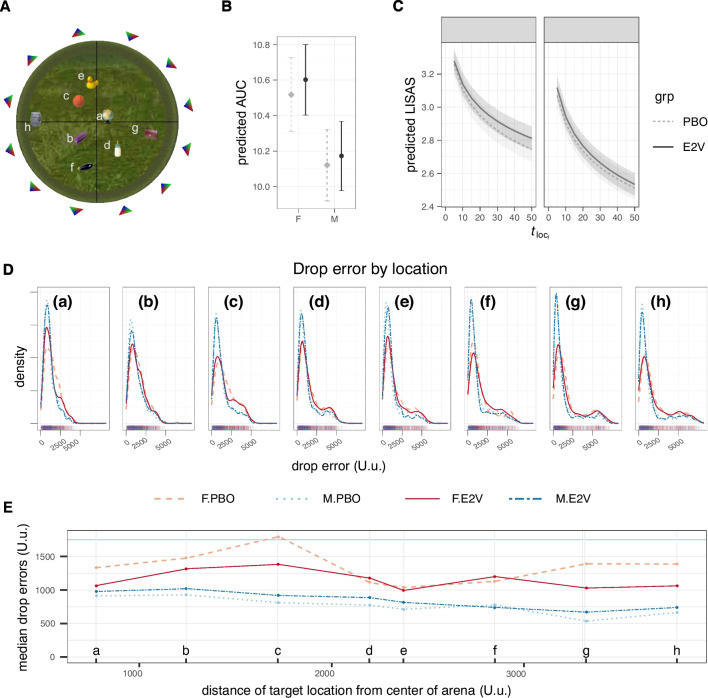
Spatial memory performance in the arena task as an indication of allocentric spatial navigation ability. **A** Aerial view of the open, circular arena marked with the same eight locations participants had to associate with eight everyday objects. Object-location pairings were randomized across participants. The only orientation cues were the grassy banks of the arena and 12 triangles hanging equally spaced in 360^∘^ on the horizon. The change in the orientation of the triangles imposed a reference axis on the arena. Speed-accuracy trade-off in terms of **B** area under the curve (AUC) of navigation duration to drop-off error, and **C** the Linear Integrated Speed-Accuracy Score (LISAS), both indicated that men were more efficient at dropping off objects at their correct locations. LISAS indicated that task efficiency increased over time, with F.PBO becoming more efficient than F.E2V with time. Error bars/areas are standard errors. **D**, **E** Memory performance was measured as the Euclidean distance between where an object was dropped off and its actual location. The maximum theoretical drop error was 10,000 Unreal units (U.u.), the diameter of the arena. **D** The distribution of the average drop errors by group and **E** median drop errors as a function of distance between the target object location and the center of the arena: women on placebo (F.PBO; dashed/light red lines) or estrogen (F.E2V; solid/darker red lines) and men on placebo (M.PBO; dotted, light blue) or estrogen (M.E2V; short-long dashed/dark blue lines), across the 8 locations, plotted from the most central location (left panel, a; plot panel labels correspond to the location labels in **A**) to progressively more peripheral locations (going towards right panels, from a to h). Drop errors by location were similar in the same sex groups (no difference between treatment groups), with men performing better than women across locations. The fluctuation of drop errors due to location followed a similar pattern in both sexes: errors were generally lower for items closer to the edge of the arena aside from location a which was close to dead center. However, unlike men, women seemed to also have been influenced by the polar axis and mirror placements more, which counteracted the ease of distance estimations for objects located near the edge of the arena

The training block ended when participants reached the end of the target corridor five consecutive times, after which the probe trial began when participants were placed in the third arm of the maze. Once they reached the end of any of the other two arms of the maze, the game ended with no feedback sound. Participants moved at their own pace, and the task was not timed. Time required for game play varied but was on the order of several minutes (less than 10 min).

If participants followed the same sequence of turns during the probe trial as they did in the training block, they were considered to have navigated egocentrically. Otherwise, if they noticed the change in their location and found the target location following the distal cues, they were scored as having adopted an allocentric strategy. Those who expressed noticing a change of landmarks in the probe trial (“Did you notice any changes in the environment?” in post-task follow-up) but continued to follow the same sequence of turns, often because they assumed that the room decoration had changed and not their starting point (ego$+$ in Fig. 2D), were excluded from analysis.

The Y-maze is a simple environment widely used in animal studies where a binary decision indicates participant’s tendency towards allocentric or egocentric navigation strategy. Although simple and based on a single probe trial, it has been effectively used to determine strategy in rodent studies (Dohanich 2002) and human studies (Parizkova et al. 2018; Rodgers et al. 2012). Of note, the Y-Maze variant we used was equivalent to the paradigm used in rodent studies where allocentric performance increased with estrogen (as reviewed in the Introduction; also both r.SD: Korol et al. 2004; Velázquez-Zamora et al. 2012).

### Arena

The arena task complemented the Y-maze in permitting a more multifaceted investigation of navigation biases. It additionally allowed us to quantify the strength of spatial representation and orientation and skills requiring an allocentric strategy. This task, previously developed and reported elsewhere (Navarro Schröder et al. 2015; Nau et al. 2020), is an object-based spatial memory task in an open circular outdoor environment inspired by the classic MWM used in animal studies (Morris 1981). Like the Y-maze, it is a commonly used environment to test the effects of hormones on learning and memory but affords more complex characterization of navigation behavior (Dohanich 2002). The arena task used here critically shares the same minimal navigation cues in the MWM as the only proximal cues in the environment are the banks of the arena: localization must be made based on distance calculations between a target object in the environment and the banks and extramaze cues, therefore favoring allocentric navigation (Rogers et al. 2017). Performance differences between men and women have been previously reported in a version of this task, with men performing better than women, and better performance associated with increased hippocampal activity (Kunz et al. 2015).

Participants could freely move within the grassy circular arena, of radius 5000 Unreal units (U.u.; equivalent to about 16 virtual meters) delimited by grassy banks, at a fixed speed using keyboard buttons. In the first phase of the task, they were instructed to remember the location of common, everyday objects (four on Day 1, eight on Day 2), which sequentially appeared somewhere in the arena, and navigate towards them to pick them up. The next object appeared in the arena as soon as the previous one was collected. After all objects had been seen and collected once, the game restarted for the recall phase. At the very start of the game, participants were placed in a rather central location (879 U.u. from the center). A picture of one of the objects would appear at the top of the screen for 2 s to prompt participants to bring the object shown to its location. This was done at the participants’ own pace. As soon as the object was dropped off, a graded feedback of how close the object was placed from its correct location was given in form of a smiley which appeared for 1.5 s on top of the screen. Five grades of feedback were given from smiling and green for within a 700-U.u. radius (about 2.5 virtual meters) of the correct location, to unhappy and red for disparities greater than 4500 U.u. The object would then appear again in the correct location, which participants had to navigate towards to pick the object up (re-encoding) before they would be cued the next object.

To familiarize participants with navigating in the arena without possible visual and memory confounds from the training session, the Day 1 training arena had different reference cues and objects than the actual test arena on Day 2. On Day 1 when participants were briefly introduced to the task, the only cues were mountains in the distance, and the locations of four common everyday objects were to be remembered. After collecting the objects once, participants dropped each object off once.

During the actual testing post-E2V/PBO treatment on Day 2, the extramaze cues were twelve rainbow-colored triangles equally spaced on the horizon spanning the full 360^∘^ instead of mountains. Six triangles on one half of the sky pointed upwards and the other six pointed downwards (Fig. 5A and Fig. 3A), thus creating a polar reference axis for the environment. As before, the distance of object locations to the banks served as the sole intramaze cue, and otherwise, object locations had to be related to the distal cues (here, the triangles on the horizon). In this arena, orientation required participants to notice the polar axis of the arena delineated by the triangles. Participants had eight common, inanimate objects different to those seen on Day 1. Like on Day 1, they collected the objects once. They then dropped off objects in 6 blocks of at least 10 min (rather than limiting game play of each session to a strict 10-min limit, they were allowed to finish a drop-off that was not completed before the 10-min mark). All participants were presented the same 8 objects and tested on the same 8 locations, but object-to-location pairings were randomized across participants. During memory recall, the order of objects cued were randomized within blocks of the complete 8 locations. Therefore, the number of times an object had been repeated in any given time block differed between participants at most by one. The recall phase on Day 2 was performed in an MRI scanner, the results of which are not reported here.

### Town

The town task, developed in Unity3D, allowed us to study the transition from egocentric to allocentric strategies in a feature-rich, complex, and highly realistic environment. It had been previously used to study the effect of cognitive map consolidation on allocentric pointing (*dead reckoning*; Craig et al. 2019). In order to facilitate allocentric referencing while moving around the town, two constantly visible tall structures (a crane and a TV tower) were placed at strategic locations (blue dots in Fig. 4C).

The task was split into two phases: route learning and pointing. Participants were informed only about the first (route-learning) phase. They were instructed to memorize a route through a town which they would be taken along as a passenger in a taxi. They were advised to take note of six buildings (e.g., a hotel) without further elaboration, which they were shown images of before route learning. The town had a total of 16 intersections. The 6 landmarks were all located at intersections in such a way that they could not be directly seen from each other (critical for the second phase).

During route learning, participants were moved passively through the town in first-person view at a constant speed (2.8 m/s) on a fixed route (red dotted line in Fig. 4C), as if they were sitting inside a taxi, pausing for 4 s at each of the six landmark intersections. After two such learning runs, a probe run started in which the player was moved along the same route through the town but stopped at each of the 16 intersections. At these intersections, green arrows indicating all the possible directions to continue then appeared on the ground, and participants had to indicate the direction they had been shown in the previous two runs using arrow keys on a keyboard (up arrow for straight, left arrow for left turn, or right for right). The color of the arrows turned red as feedback for wrong answers. Irrespective of whether the response was correct, the game then continued along the correct route. If participants made any errors during this probe run, they had to complete a learning cycle (one learning run followed by a probe run) until they committed no turning error during the probe run.

Upon successful completion of the first phase, a surprise pointing test began. During this second phase, participants were placed at each of the 6 landmarks and were asked to rotate the player viewpoint to face the direction where a cued landmark is located using the right and left arrow keys on the keyboard. Participants could take as much time as they wanted for their responses, and no feedback was given once they confirmed their answer by pressing the keyboard space bar. Trial order (total 30 trials, testing all possible pairwise combinations twice) was pseudo-randomized across participants.

## Analyses

We expected performance differences between men and women on these spatial tasks and the influence of E2V to vary depending on sex. Therefore, unless otherwise specified, full regression models and ANOVAs (type III, R package afex) refer to models where the specified dependent measure was regressed on sex, treatment group (E2V or PBO), and their interaction, in that order for Wald tests. All analyses were done in the R statistical environment and plots shown generated with the R package ggplot2 (Wickham 2016). Likelihood-ratio chi-squared tests (LRT) of nested regression models (in part calculated with the R package lrtest for linear mixed effects models), stepping backwards from a fully specified model, was used to judge which model described the data more aptly. Alternatively, the difference in the information criteria AIC ($\Delta$AIC) or BIC ($\Delta$BIC) was used for model selection, notably where model comparisons were not nested. Models were visually checked for residual homoscedasticity, normality and fit (facilitated by several R packages for model visualization, summary, and diagnostics including emmeans, jTools, modelsummary, sjPlot, and vcdExtra). Mean model parameter estimates with [95% confidence intervals] are reported with associated Wald tests, which indicate the order-dependent marginal contribution of the parameter, or ANOVAs of individual parameter contribution to reducing the residual sum of squares of models.

### Wayfinding and navigating biases

#### Y-maze

Bias towards egocentric or allocentric strategies due to sex and/or treatment, as assessed by the Y-Maze task, was modeled with logistic linear model of the number of allocentric navigators in the four groups. If E2 serves to enhance spatial navigation, we would expect the E2V groups to be more adaptive. In this task, flexibility is noticing location change during the probe trial and navigating allocentrically. Based on previous research, we might expect more men to adopt an allocentric strategy, which might be more pronounced in men on E2V, and given the nonlinear E2V-dose response, that the tendency in women to navigate egocentrically on such tasks to be even more pronounced on E2V.

### Egocentric strategy ability

#### Arena

The arena task complemented the Y-maze by offering a means of investigating finer differences in navigation biases. As the only proximal cues in the arena were the banks demarcating the arena boundaries, the open arena prevents egocentric navigation; nonetheless, the task can be completed through egocentric strategies, random strategies, or unsystematic navigation. Therefore, this task affords a more complex analysis of the spatial patterns of navigation, including that of the finer characteristics of egocentric navigation (allocentric navigation strategies described further below).

The strategy of navigating close to the boundaries of an open arena and avoiding more central areas (*thigomataxis*) is often seen in animals such as rodents, and is associated with higher stress and when animals first explore novel environments (m.B6J-WT: Rogers et al. 2017). In humans, a similar association was found (Kallai et al. 2007). Thigomataxis has also been found in healthy adult humans genetically at risk for Alzheimer’s (Kunz et al. 2015), who showed a bias for navigating more in the periphery compared to those not at risk, and is predictive of worse spatial memory performance (Kunz et al. 2015). Although navigating more in the periphery does not always lead to worse performance, it is considered less precise and its persistent use prevents allocentric learning (Kallai et al. 2007), and therefore, an analysis of this spatial behavior over time would also be informative for allocentric navigation. We therefore investigated any center/periphery preferences in two forms of analyses.

We first measured center/periphery navigation preference by tallying samples logged as the proportion of movement in the outer half (periphery) of the arena to total movement samples. If E2V enhances spatial orientation, we might expect the E2V groups to not avoid navigating towards more central areas of the arena where distance estimations are more difficult. To investigate whether this measure was influenced by E2V administration and how that differed between sexes, we fitted beta regression models with a probit link function (R package betareg, Cribari-Neto and Zeileis 2010) on this periphery navigation preference measure, calculated across the entire task and also only during object retrieval, that is, when participants navigated from the point where they dropped off an object, to the correct location to retrieve the object during the re-encoding period that directly followed every smiley feedback. Looking at object-retrieval only phases excluded the confound of object memory: as target object locations where shown, this allowed us to measure whether participants relied on peripheral/boundaries to re-encode locations irrespective of memory.

We also compared spatial patterns of navigation using heat map analyses for a more detailed cumulative view of spatial preferences over time. Heat map differences of trajectories between groups were calculated using the R package mousetrap. Mean trajectory density difference maps were created by first smoothing path crossings in a given region by a 5-standard-deviation Gaussian, low-pass filtering, normalizing, and then nonlinearly transforming intensities (log). As heat maps are subject to binning effects, the temporospatial differences between trajectory maps were quantified using the autocorrelation metric, the local Getis-Ord statistic, Getis-Ord^∗^ ($G_i^*$, Getis and Ord 1992; Ord and Getis 1995). The $G_i^*$ statistic is a z-score of the concentration of a given spatial feature in weighted centroids within a specified distance from each point, including the point itself. A positive $G_i^*$ here indicates local spatial clustering of path crossings. To calculate the Getis-Ord $G_i^*$, the arena was first divided into hexagonal tiles, which optimally tessellate a circular environment. K-nearest-neighbors was used to find the most appropriate neighbor size for the centroid of each hexagon, and the ratio of path crossings in a hexagon to its neighbors was taken and standardized (R package spdep, Bivand and Wong 2018). The $G_i^*$ statistical maps shown (Fig. 3) are thresholded at $p < 0.10$.

#### Town

Although the arena provides an unconstrained environment to probe navigation behavior, it is not necessarily ecologically valid for humans. We therefore also included the town task which more closely reflects real-world navigation with more visually complex environments and routes (Ekstrom and Isham 2017; Ruddle and Lessels 2006).

The first part of the town task focused on retracing a route, which could be solved by an egocentric strategy. As this part of the task was relatively easy for most, we would not necessarily expect sex or treatment differences in the two heuristic measures used here: (1) the number of rounds through the town needed until the participant was able to retrace the entire route with no errors (a minimum of two rounds even if participants made no error in retracing the route the first time around), and (2) the cumulative number of errors made along the route across all rounds. Most participants were able to retrace the route through the town with no errors within the first two rounds, but the data was not overdispersed, so Poisson regression modeling was used. The total number of errors made in retracing the route was highly overdispersed (dispersion ratio 3.37 of a Poisson model, Pearson’s $\chi ^2 = 421.7$), with 8.5–12.4% of participants in each group able to retrace the route without any errors (and thus completed this task in the minimum two rounds). Rootograms of quasi-Poisson model predictions, or square-root histograms of the total wayfinding errors plotted hanging from the curve of model predictions (R countreg package, Kleiber and Zeileis 2016), indicated bad model fit characteristic of data overdispersion not only due to zero-inflation, at least handled by quasi-Poisson modeling, therefore indicating that a negative binomial (NB) model might be more appropriate. Zero-altered negative binomial (ZANB; also called truncated or hurdle) models were thus fitted (hurdle function from the R package pscl, Zeileis et al. 2008) and compared using Vuong tests for non-nested models (R package nonnest2). ZANB models zero mass with a binomial distribution and logit link separately from positive counts, which are modeled by a truncated negative binomial distribution. This therefore permits inferences about factors contributing to making no errors (zero mass) independently from making errors (positive counts).

### Allocentric strategy ability: cognitive spatial maps and spatial memory

The arena and town tasks also allowed us to explore any differences between the sexes or due to elevated E2 levels in the ability to create spatial cognitive maps and to adopt allocentric navigation strategies.

#### Arena

The 3-D computer-simulated arena had no intramaze cues — spatial orientation necessitated distance estimation between target objects and the banks of the arena, and the use of the extramaze cues on the horizon. Spatial orientation in a space with no proximal cues and accurate distance estimation are abilities supporting allocentric navigation, a strategy which is more strongly associated with the ability to build cognitive maps (Tolman 1948). Performance in the virtual arena thus gave us a means to investigate strength of allocentric navigation abilities. This was done in terms of object-location accuracy and time participants took to drop off objects at their designated locations. We expected the men on E2V to develop a stronger sense of spatial orientation more quickly as would be indicated by a faster learning rate (fewer errors over time) and be less constrained by the minimal cues in how they moved through the arena and hence more efficient in their navigation.

##### Complete object drop-offs (trials)

As the task was self-paced with no time pressure within the limits of 6 10-min sessions, total number of objects dropped off over time gave some indication of spatial navigation certainty. Although certainty does not always imply better memory, better spatial memory performance tends to be associated with shorter recall times (Burgess et al. 2002). We looked at how the number of objects dropped off changed over the entire task (1 h), across 10-min blocks of time (duration of each run), and blocked by the number of times a particular object (and hence location) was seen. We regressed each on sex, E2V treatment, and their interaction with NB models because of data overdispersion (trial-by-trial data: dispersion ratio 14.7, $\chi ^2 = 1841.7, p<0.001$; 10-min averages: dispersion ratio 3.1, $\chi ^2 = 2349.4, p < 0.001$). However, rootograms showed that NB models still fitted number of overall objects dropped off poorly. A complement to the total number objects dropped off is the total number of repetitions of each object. As object cueing was randomized within sets of the complete 8 objects, the number of object repetition should remain similar across all object locations at any given point in time. However, although this data was also overdispersed (dispersion ratio 1.8, $\chi ^2 = 1849.8, p < 0.001$), rootograms showed that NB models on repetition times of object locations fitted the data much better than those of overall total number of trials/objects dropped off, which also suggests that the object locations differed in difficulty and should therefore be considered.

Alternative to the number of objects dropped off over time was the time spent for an object drop-off. Several time measures characterizing object drop-off were taken: (1) the number of drop-offs exceeding 1 min (i.e., number of 1-min intervals with no drop-offs) modeled with zero-inflated Poisson (ZIP) models, and (2) the length of time participants took to drop off an object (navigation duration).

Scatterplots of time for each object drop-off (navigation duration) showed an exponential decay over time, which was linearized by taking the log of navigation duration. The overall differences in navigation duration between groups were tested in a linear model with log-transformed navigation duration regressed on sex, treatment group and their interaction. A finer mixed linear model was also performed (R package nlme, p-statistic estimated with R package car’s function anova which performs a type III test on model deviance) with temporal autocorrelation defined by a first-order autoregressive structure (AR1), which estimates a regression coefficient between subsequent residuals within each individual, and an alternative model with autoregressive moving average (ARMA) also specified to vary by individual were fitted to the log of navigation duration regressed on time indexed by the repetition of a location, E2V treatment, sex, centered object drop-off accuracy (drop error), and their interactions. As the 8 object locations were distributed through the arena in order to sample a wide range of locations, this also resulted in locations that might have varied in difficulty depending on individual spatial ability (e.g., central locations might be more difficult for some as they require finer distance estimates); therefore, a random term was specified to allow for different relationships in navigation duration with these locations for each individual. The estimates from modeling with AR1 correction was similar to ARMA correction. Although both models had heteroscedastic residuals (funnel-shaped, increasing residual variance over time), estimates are nonetheless reported here from the full model with AR1.

##### Memory accuracy: drop errors

Successful cognitive mapping is more directly assessed by spatial memory performance in the arena. Memory performance was indexed by object drop-off accuracy (drop error), calculated as the Euclidean distance in U.u. between where a participant dropped off an object in the arena and the correct location.

Navigating time to drop off an object traded off with drop error. We therefore combined the two measures into the integrated scores: (1) area under the curve (AUC) of drop errors over navigation duration (the higher the AUC the worse the performance), and (2) a pointwise variant of the linear integrated speed-accuracy score (LISAS: Vandierendonck 2017) considered over time. LISAS can be considered as RT with an error penalty: the higher the LISAS, the less efficient the performance. The LISAS used here was calculated as $RT_{trial_i} + \frac{SD_{RT}}{SD_{DE}} DE_{trial_i}$, where $RT_{trial_i}$ is the navigation duration for an object-location drop-off trial, and *DE* is the associated drop error weighted by the ratio of the standard deviations (*SD*) of the participant’s *RT* to *DE* across all items.

Log-transformed AUC was regressed in a linear mixed model on sex and treatment group with intercepts allowing to vary by participant. The log-transformed LISAS score was regressed on sex, treatment, the log-transformed object-location repetition number (as a proxy of time points), and their interactions, in a mixed effects linear model, where slopes were allowed to vary for different object locations by participant.

##### Movement patterns

In addition to quantifying heat maps of movement in the arena to investigate spatial clustering of movement (see the “Egocentric strategy ability” section), we also considered various additional movement and trajectory metrics to evaluate movement efficiency, as indicators of sense of orientation. As the arena task involved directed paths towards target locations, deviations from the straight paths from navigation start points to target locations could be indications of lower orientation efficiency (Benhamou 2004). We would expect increased E2, as an enhancer of allocentric navigation, to decrease such deviations. Several metrics (with respective models tested) were considered to capture this deviation: total distance covered per path between target locations (log-normal), maximum absolute deviation from a straight path, sinuosity, i.e., ratio of distance traveled to as-the-crow-flies distance (log-normal), and number of direction changes along the x- and along the y-axes, i.e., x-/y-flips (Poisson regression). We also considered idle time (log-normal) to quantify periods of movement inactivity, arguably indicative of deliberation and hence uncertainty in spatial orientation.

These metrics were calculated for the 36 possible trajectories (i.e., $$\left( {\begin{array}{c}9\\ 2\end{array}}\right) $$ for the pairwise combination of 8 locations plus initial starting point at the very start of the game, not considering direction of paths). The R packages used to calculate the metrics were sp (Bivand et al. 2013), sf (Pebesma 2018), amt (Signer et al. 2019), and mousetrap (Kieslich et al. 2019). The average of each metric except for sinuosity and x-/y-flips, was then modeled (with the model variant already indicated above) using mixed linear effects models on sex, group and their interaction (in that order), allowing intercepts to vary for each participant and for the different paths. Sinuosity models differed in random terms which allowed intercepts to vary for each participant and slopes of different paths by participant. Direction changes on the x- and y-axes were underdispersed (x-flips: dispersion ratio 0.89, $p<0.001$; y-flips: dispersion ratio 0.9, $p<0.001$) and were therefore fitted with generalized linear Conway-Maxwell-Poisson models.

**Table 1:** General overview of navigation (nav) behavior differences between the sexes (men M; women F), with estradiol valerate (E2V) or placebo (PBO) administration (treatment), or that were non-additive (sex $\times$ treatment) in the respective tasks

			Group differences		
			Sex	Treatment	Sex $\times$ Treatment
**Egocentric strategy**					
		Egocentric bias ^(Y)^	F>M	*n.d.*	*n.d.*
		# route attempts ^(T)^	*n.d.*	*n.d.*	*n.d.*
		# route errors ^(T)^	*n.d.*	*n.d.*	F.E2V>F.PBO
		Idle time ^(O)^	F>M		
		Drop-off time (nav dur) ^(O)^	F>M	E2V>PBO	F.E2V>F.PBO
		Periphery preference ^(O)^	F>M	*n.d.*	*n.d.*
**Allocentric strategy / cognitive map development**					
		Pointing accuracy ^(T)^	M>F	*n.d.*	*n.d.*
		Quicker pointing speed ^(T)^	*n.d.*	*n.d.*	F.PBO>F.E2V
		Total # drop-offs ^(O)^	M>F	–	F.PBO>F.E2V
		Speed-accuracy (1/LISAS) ^(O)^	M>F	*n.d.*	F.PBO>F.E2V over time
		Nav confidence (nav in confident locs) ^(O)^	–	E2V>PBO (central)	F.E2V>F.PBO (target locations)
**Navigated path complexity**					
		Traveled distance ^(O)^	M>F	*n.d.*	*n.d.*
		Sinuosity ^(O)^	M>F (+ over time)	PBO>E2V	*n.d.*
		# x-/y-flips ^(O)^	M>F	*n.d.*	F.E2V>F.PBO, M.PBO>M.E2V

Metrics are framed so the greater the value for the metric, the greater the bias towards the strategy the metric is listed under. Therefore, the group with higher values (left of the greater sign, “>”) demonstrated greater possibility of the given strategy, at least according to the given metric

^(Y)^ Y-maze, ^(O)^arena, ^(T)^town task

*n.d.* = no differences. dur = duration, locs = locations, RT = response time

#### Town

Allocentric strategy assessment was done with the following two measures from the town task: participant mean accuracy in pointing towards the location of landmarks (pointing accuracy) and mean response time taken to indicate this orientation (pointing RT). Pointing accuracy (i.e., *dead reckoning*) is considered an indicator of path integration and of a developed cognitive map, the basis of an allocentric strategy (see the “Introduction” section). Some previous studies have found men to be more substantially more accurate than women in pointing accuracy, and that in both sexes, errors increased with the number of navigation turns made (in a 3-D maze: Lawton and Morrin 1999). We assessed pointing accuracy by calculating the absolute difference in degrees between the correct direction and the direction indicated by the participant. E2V as an enhancer would be expected to lead to more accurate and quicker pointing responses. A generalized linear model with a log link (Gamma distribution) was fitted since mean pointing error is always positive and also positively skewed.

As is often the case for response times, the distribution of pointing RTs was right-skewed. An inverse Gaussian model with the link function $1/\mu ^2$ was used to mitigate the skew in the data distribution and non-normal distribution of model residuals.

## Results

### Hormone levels

We managed to test participants in all groups about the same time of day (testing lasting an entire afternoon/through early evening), thus minimizing diurnal fluctuations in sex hormone levels (Fig. 1B). As designed, pre-treatment/Day1 E2 concentrations did not differ between sexes (and/or randomly assigned groups; highest $F_{1,125} = 1.77, p=0.19$). Testosterone (TST) was the only sex hormone measured in saliva that differed between sexes (TST sex difference $F_{1,125} = 161.45, p < 0.001$; no difference between groups or interaction with sex, highest $F_{1,125} = 0.2, p = 0.66$; P4 highest $F_{1,125} = 0.59, p=0.44$).

E2V administration increased E2 levels in the E2V group compared to the PBO group (ANOVA indicating group difference $F_{1,119} = 245.76, p < 0.001$). This increase was higher in the male than female E2V group (ANOVA group $\times$ sex: $F_{1,119} = 8.94, p < 0.005$; post hoc Tukey tests indicate only a difference between F.E2V and M.E2V, $p < 0.001$, and not for F.PBO vs. M.PBO, $p = 1$). E2V administration also lowered TST levels in M.E2V (ANOVA group $\times$ sex: $F_{1,125} = 25.48, p < 0.001$) to levels lower than M.PBO ($p<0.001$) but was still higher than that of F.E2V ($p<0.001$). P4 levels remained the same between the sexes following E2V administration (ANOVA group $\times$ sex: $F_{1,125} = 4.55, p < 0.05$; post-hoc Tukey tests indicated no difference in P4 levels between the E2V groups: F.E2V vs. M.E2V, $p=1$, and that it was higher in women than men in the PBO group, $p < 0.05$).

### Wayfinding/navigating biases

A general overview of the results across all tasks is summarized in Table 1.

#### Y-maze

There was no bias towards egocentric or allocentric strategies between E2V and PBO groups, as indicated by behavior on the Y-maze task. More men tended to adopt an allocentric strategy (LRT $\chi ^2_{1} = 2.83, p = 0.093$ in favor of a logistic model with only sex as a predictor, but this explained less than 1.5% of the data variance: adjusted $R^2 = 0.014$).

#### Arena

Overall, central or more peripheral navigation in the arena task was consistent with Y-maze behavior. E2V treatment did not contribute to the prediction of less peripheral navigation, but there was a difference between the sexes with women tending to navigate in the periphery of the arena compared to men (LRT better-fitting beta regression model only indicated sex as a factor: LRT $\chi ^2_{1} = 3.30, p<0.069$ over a null model; $Pr(\texttt{periph}_{F}=1 \text {|} {\textbf {X}}) = \Phi (- 0.99 - 0.052*\mathtt {Sex_M})$, Sex: 95% CI $[-0.11, 0.0038]$, Wald $z = -1.83, p = 0.068$; precision estimate $\Phi = 84.88, z = 8.05, p < 0.001$). However, this model only explained less than 3% of data variance (pseudo-$R^2=0.027$) and residuals were slightly bimodal (as was also the case with other link functions tested — logit, probit and Cauchit — but which generated similar parameter estimates). Women’s tendency to move more in the periphery than men was clearer during object retrieval (best-fitting beta regression model with probit link function had sex as the sole predictor; LRT $\chi ^2_{1} = 10.15, p<0.005$ over a null model; $\mathtt {Pr(periph_F} = 1 \text {|} {\textbf {X}}) = \Phi (-1.0-0.11*\mathtt {Sex_M})$, Sex: 95% CI $[-0.17,-0.04]$, Wald $z = 8.05, p < 0.001$; with pseudo-$R^2$ = 0.078 and precision estimate $\Phi = 65.77, z = 8.05, p < 0.001$). This model predicted that during object retrieval, the odds of more allocentric navigation behavior were 1.08 higher than in men.

Heat maps (Fig. 3) qualitatively suggested that E2V groups seem to navigate more centrally in the arena regardless of sex. This was confirmed by $G_{i}^*$ statistical maps (Fig. 3, bottom panels). Specifically, the differences in spatial clusters between the male groups were more compactly focused around target object locations than between the female groups. Compared to PBO groups, there was also more spatial clustering in the central regions of the arena for E2V groups compared to the respective PBO groups, a difference which was particularly more pronounced between the female groups.

### Egocentric strategy ability

#### Town

Most participants in all four groups were able to retrace the route through the virtual town with no errors during the minimum two test rounds through the town: 90.0% F.PBO, 82.8% F.E2V, 84.4% M.PBO, 85.3% M.E2V. All participants were able to retrace the route with no error by the fourth round except for one man who did it by the fifth. The number of rounds needed to complete a full round with no turning errors did not differ between the sexes, E2V treatment, or their interaction (LRT of Poisson regression models favoring a null model compared to regression on sex $\chi ^2_1 = 0, p=0.98$ or on treatment $\chi ^2_1 = 0, p=1$).

This was also the case for the total number of turning errors made, summed across all rounds completed (LRT of ZANB models favored the null model over a model with sex only, $\chi ^2_1 = 0.53, p=0.77$, or treatment group only, $\chi ^2_1 = 2.2, p=0.33$). No errors at all were common: 53.3% F.PBO participants, 33.3% F.E2V, 50.0% M.PBO, and 44.1% M.E2V. ZANB models suggest that sex, treatment and their interaction do not clearly explain the errors (all Wald tests $p>0.2$, $\Delta$AIC = 1.8 favoring a null count model). However, the odds of women on E2V (F.E2V) making any errors at all compared to women on PBO (F.PBO) were 229% higher (exponentiated zero-mass est. 0.83 [0.43, 3.11], $z=1.59, p = 0.11$; Fig. 4B); full zero-inflated model predicted the incidence odds ratio of making no errors for F.PBO $= 0.88 + 1.14 * \mathtt {Sex_M} + 2.29 * \mathtt {Grp_{E2V}} + 0.55 * \mathtt {Sex_M:Grp_{E2V}}$, adjusted $R^2 = 0.088$; 95% CI: intercept/mean zero errors in F.PBO [0.43, 1.79] $p = 0.72$, Sex [0.42, 3.10] $p=0.79$, Grp [0.83, 6.33] $p = 0.11$, Sex:Grp [0.14, 2.26] $p=0.41$).

### Allocentric strategy ability: cognitive spatial maps and spatial memory

#### Town

Pointing errors were slightly bimodal among women and right-skewed in all groups. Generalized linear modeling of pointing errors modeling pointing errors during egocentric landmark localization with the gamma distribution predicted men to make smaller errors (Fig. 4D; mean estimate 11.5 smaller magnitude in pointing error in M.PBO than F.PBO, 95% CI [02.23, 0.027]; LRT also confirmed that the better-fitting model only included sex as a factor: $\chi ^2_1 = 10.45, p < 0.005$, with similar estimates from the reduced model: overall men had 11.0 $[-18.10, -3.90]$ smaller pointing errors than women, Cragg-Uhler pseudo $R^2 = 0.078$).

Sex did not explain pointing RT, however, according to inverse Gaussian linear models. There was some indication that F.E2V were slower than F.PBO (Fig. 4E; est. $-0.018$ $[-0.0040,0.0030]$, Wald $t = -1.72$, $p = 0.087$, with similar estimates for a reduced model with only treatment group as a factor: $-0.0012$ $[-0.0027, 0.0020]$, Wald $t = -1.63$, $p = 0.11$, reduced model Cragg-Uhler $R^2 = 0.020$, LRT against a null model $\chi ^2_1 = 2.56, p = 0.11$).

#### Arena

The same 8 drop-off locations were used across all participants, but the object associated with each location was randomized among participants. Participants repeated drop-offs for an object/location a median of 23 times (mean±SD = 23.3 ± 7.1). Overall, M.PBO dropped off a predicted $e^{0.2} = 1.2$ [1.1, 1.4] times more objects over the 1-h arena task than F.PBO (Wald $z = 2.92, p < 0.005$) and a bias for F.PBO to drop off $e^{0.90} = 2.5$ [0.8, 1.0] times more objects over the 1-h period than F.E2V (Wald $z = -1.51, p = 0.13$), with no other reliable biases, based on a full NB model which captured 17.8% of data variance (Cragg-Uhler pseudo-$R^2$; $\Delta$AIC = 1.5). The better-fitting ZB model on the analogous measure of repetition times of object locations also indicated that a full model with sex, treatment and their interactions captured about 16% of the variance. This model indicated that there was a greater difference in number of repetitions between the sexes, with men completing drop-offs of the same objects more often (Wald $z = 8.4, p < 0.001$), and also due to treatment, with the E2V groups not completing as many of the same objects overall (Wald $z = -4.2, p < 0.001$), but that these effects varied depending on sex (interaction sex $\times$ treatment group: $z = 2.4, p < 0.05$; LRT favoring the full model with interaction $\chi ^2_1 = 5.8, p < 0.05$, $\Delta$AIC =3). M.PBO were predicted to complete marginally more repetitions of the same objects than M.E2V, but the difference is markedly greater for F.PBO who repeated considerably more objects than F.E2V.

A complement to dropping more objects is being faster in bringing objects to where drop-off points were thought to be. This was indeed the case. As would be expected, as men were able to drop off more objects, they were also considerably quicker at dropping off each object (navigation duration) than women overall ($t = -9.3, p < 0.001$), while in general the E2V group took longer ($t = 16.1$, $p < 0.001$), though this was driven by the slower times in the F.E2V compared to the F.PBO, as there were no predicted differences between the male groups (interaction sex $\times$ treatment: Wald $t = -12.3, p < 0.001$; overall full model fit $R^2 = 0.037$, LRT favoring the full model $p < 0.001$: log of navigation duration in F.PBO = 2.38 - 0.094 * Sex[M] + 0.17 * Grp[E2V] - 0.18 * Sex[M]:Grp[E2V], 95% CI Intercept/mean F.PBO [2.36,2.39] $t=313.17$ $p<0.001$, Sex [$-$0.11, $-$0.07] $t=-9.32$ $p<0.001$, Grp [0.15, 0.19] $t=16.12$ $p<0.001$, Sex:Grp [$-$0.20,$-$0.15] $t=-12.35$ $p<0.001$).

Spatial memory accuracy was calculated in terms of the Euclidean distance between where an object was dropped off and its actual location (drop errors). Drop error generally decreased exponentially over time in all groups, but there was great interindividual variability. Drop error plots across object locations (Fig. 5) also showed a small bump of drop errors greater than 5000 virtual units (180^∘^) notably for peripheral locations. These distribution plots indicate that there were no location-based differences between E2V and PBO groups. Men performed better than women across locations, but the change of drop errors due to location followed a similar pattern in both sexes: errors were generally lower for locations closer to the edge of the arena (more smaller drop errors) aside from the location close to dead center of the arena (location (a) in Fig. 5D). However, whereas the density of low drop errors in men increased monotonically as locations approached the banks of the arena, the density of lower drop errors in women was high only up to a certain distance and then decreased again so that locations for objects closest to the banks of the arena were comparable to the drop errors for the more central locations. To illustrate, one of the most peripheral locations (f), had one of the highest densities of low drop errors among men but the lowest densities of low drop errors in women.

The AUC of navigation duration to drop-off error (Fig. 5B and Suppl. Table S1) did not show differences in treatment between the sexes (LRT and Wald $t = 0.15$) or an overall treatment effect (LRT and Wald $t = 0.57$). Women, however, had higher AUC than men overall (LRT favoring model with only sex as a factor $\chi ^2_1 = 15.7, p<0.001$; full model Wald $t=-4.06$ $p<0.0001$).

Similarly, speed-accuracy trade-off in terms of LISAS (Fig. 5C and Suppl. Table S1) was more efficient (lower) in men than women (Satterthwaite $F_{1,137.9} = 23.1$, $p < 0.001$). Additionally, LISAS changed with time regardless of group (Satterthwaite approximation $F_{1,23010.6} = 6043.6, p < 0.001$). Treatment difference was only apparent in women over time where LISAS were similar in the beginning in both female groups, but with time, women on PBO became faster while maintaining similar accuracy compared to women on E2V (Satterthwaite $F_{1,23010.6} = 8.0, p < 0.005$, LRT $\chi ^2 = 6405.9, p < 0.001$, $\Delta$AIC = 6336).

#### Arena movement characteristics

##### Total distance

Men traveled 18% longer paths between target locations, i.e., between a pick-up location (re-encoding) and where they dropped off the next cued object (Suppl. Table S1; Wald $t=2.32$, $p<0.001$).

##### Idle time

In general, men paused 45% less than women during navigation (idle time, Suppl. Table S1; LRT $\chi ^2_1 = 1.35$)

##### Path complexity

There was no difference in maximum absolute path deviation by sex or treatment (LRT favored a null model over a single sex regressor $\chi ^2_1 = 0.32$, $p = 0.57$ or a single treatment regressor $\chi ^2_1 = 0.39$, $p = 0.53$). There was also no difference in sex or treatment in absolute path deviation (LRT favored a null model over a single sex regressor $\chi ^2_1 = 0.0024$, $p = 0.96$ or a single treatment regressor $\chi ^2_1 = 0.57$, $p = 0.45$).

Men also changed directions along x- and y-axes about 20–32% more than women overall, but E2V treatment is predicted to increase direction flipping particularly in women by 16% but to reduce direction flipping in men (LRT in favor of sex by treatment difference: x-flips $\chi ^2_1 = 22.4$, $p<0.001$; y-flips $\chi ^2_1 = 13.9$, $p<0.001$)).

Men were 26% more sinuous in their path movements than women overall, but over time, the difference diminished ($\Delta$AIC = 2 for inclusion of interactions; Suppl. Table S1, Fig. 3B). Elevated E2 was predicted to lead to path movements that were about 10% less sinuous.

## Discussion

We found sex effects to dominate any effects of elevated circulating E2. When there were behavioral changes with elevated E2, they were stronger in women and did not clearly improve allocentric performance. Instead, the female E2V group generally performed worse with allocentric navigation and better with egocentric navigation than the female PBO group, but the pattern of our results — like much of the literature whether of human or animal studies — did not consistently fall into this general trend, and we did find differences depending on sex. Such sex-dominant effects and sex-dependent responses to elevated E2 have been reported before. For example, in a test-delay (more difficult) version of the 8-arm RAM with rats, males (young, gonadectomized and old, intact) demonstrated better spatial working memory than females (young, ovariectomized and old, intact) irrespective of E2 administration, but when there was an effect on performance in females, E2 worsened performance (r.SD: Luine and Rodriguez 1994) while it improved spatial working memory learning in male rats. Similar to this study in which plasma E2 concentration in (ovariectomized) female rats were elevated to the maximum levels in the estrous cycle, the saliva E2 concentration in our F.E2V group reached the upper range of the maximum levels in the menstrual cycle. Luine and Rodriguez do not report the E2 concentration in their male rats and assumed that the levels would be lower, but we found E2 saliva concentration to be higher in our M.E2V group despite having had a lower E2V dosage than the women. Caution must be made in extending results from animal studies to humans, as problems not only stem from translating between animals and humans or unifying results across species (e.g., mice, rats, rhesus monkeys: Williams and Meck 1991), even across species of the same order such as rodents (e.g., Li et al. 2004; Manahan-Vaughan and Schwegler 2011), but also between strains of the same species (Wahlsten et al. 2005 although cf. Jonasson 2005). Nonetheless, animal studies remain an important source of inferring physiological and mechanistic causes for the differences we see.

### Men demonstrate better allocentric navigation and overall navigation flexibility

Consistent with what has been reported before in both the animal studies summarized in the Introduction and human studies (listed here), there was a slightly greater tendency for the men in this study to adopt an allocentric over egocentric navigation strategy (Y-maze) than women, which corroborates findings in some other studies (Ferguson et al. 2019; Harris et al. 2019; Hughes et al. 2014), though no simple, clear strategy differences between the sexes have also been reported on a virtual T-maze where both sexes evolved in strategy preferences with training, and eventually male participants in the study preferred allocentric navigation while female participants on average showed no preference (Astur et al. 2016).

Also similar to some human studies, men were more accurate at pointing to locations of landmarks compared to women (town: pointing accuracy; Fig. 4D; Bell and Saucier 2004; Hughes et al. 2014; Lawton and Morrin 1999; Nazareth et al. 2019, but cf. Trumble et al. 2016), ventured more away from cues in an environment (arena: path density, Fig. 3; and periphery preference measure; consistent with Devan et al., 2016), and were faster at responding to spatial memory tasks (arena: navigation duration during object drop-offs; Boone et al. 2018) in a way that did not compromise their accuracy (arena: speed-accuracy measures AUC and LISAS; Fig. 5B, C). Our speed-accuracy finding contrasts, however, with a study that found that although men were quicker in finding rewards in a virtual 8-arm RAM, they did not do so more efficiently than women (Astur et al. 2004).

Our male participants moved more sinuously than women (arena: path sinuosity; Fig. 3B and Tables 1 and Suppl. Table S1). This seems counter-intuitive based on the studies discussed thus far: we assumed that more *flexible* spatial navigation comes with better spatial orientation, that is, being able to reorient oneself and efficiently navigate to target locations regardless of starting location. Given this idea of flexibility, better spatial flexibility should result in the straightest path to target locations. On the other hand, greater sinuosity might reflect inefficient goal-directed navigation, it can also stem from spatial flexibility and confidence that facilitate greater exploratory behavior and (voluntary) deviation from known, “safe” paths (Gagnon et al. 2018; Wood et al. 2021). In a way, this is a complement to the argument that inconfident and/or less allocentric navigation will result in thigmotaxis: lack of confidence will promote conservative movements (following walls), whereas greater confidence will promote more liberal exploration. The observation that the sinuosity of navigation paths of both the men and women in our study increased over time, when all participants learned and improved on the arena task as indicated by better memory performance with time (lower drop error rates and accuracy-efficiency over time: Fig. 5C), supports this interpretation of greater sinuosity as better spatial orientation and could indicate greater confidence and/or less stress — both of which tend to be more often the case for males than females in test settings (Devlin and Bernstein 1995; Picucci et al. 2011 but also see van Gerven et al. 2016).

The arena task allowed us to further probe the extent of flexibility in spatial navigation, where the more accurate the distance judgment for greater distances from any reference points, the greater the spatial navigation flexibility, which for this task is equivalent to a more developed allocentric strategy. Using perspective judgments to estimate unequal distances to different points of highly similar visual cues in the distance (grassy banks delimiting the arena and triangles on the horizon) is generally challenging, but symmetric environments such as the arena require additionally being able to keep track of a rotational reference. Indeed, dead center of the arena (no need to keep track of rotational view, equidistant to all extramaze cues) was most accurate in both sexes, on par with the most peripheral locations for men (arena: drop error; Fig. 5B). Otherwise, the distribution of accuracy in the male groups increased linearly and monotonically as locations approached the edge of the arena, as would be expected, but this was not the case with the women. Women had a U-shaped distribution of drop errors in the arena task as locations approached the edge of the arena, in contrast to men who showed better accuracy as locations were closer to the edge. A possible explanation for this is that women used rotation-based references less, making themselves more susceptible to errors towards the periphery in such highly symmetrical environments where polar/mirror ends of the environment might be confused for each other.

### Elevated E2 lead to some spatial orientation flexibility

Despite E2’s role in neuroprotection and neurogenesis, our results indicate that the levels to which we raised E2 concentration did not lead to greater spatial navigation flexibility, that is, better sense of orientation, which permits successful reorientation regardless of non-habitual starting location in a known environment. For the tasks we have used, greater flexibility is equivalent to a clear enhancement of allocentric navigation and associated spatial memory. Our results do not corroborate what has been reported in some animal studies with either the natural, endogenous fluctuations of E2 (F-r.SD: Korol et al. 2004), exogenously elevated levels to females (hormone replacement in Fx-r.SD to about 80 pg/mL, close to the maximum level of the peak of the estrus cycle, i.e., the peak during the proestrus phase: Korol and Kolo 2002), administered to intact male mice (m.B6J: Heikkinen et al. 2002), or gonadectomized rats (r.SD: Luine and Rodriguez 1994; but cf. the review of estrogen enhancement in males Paletta et al. 2018), as well as in humans (Gillies and McArthur 2010). We found no improvement in spatial memory with elevated E2 levels in either sexes in terms of landmark pointing accuracy or spatial memory drop error. Our drop error results are in contention with previous animal studies using equivalent MWM tasks that have found, for example, that E2V administration improved performance on allocentric strategy tasks in ovariectomized rats (Fx-r.SD: Korol et al. 2004). We also did not find elevated E2 to increase idle time in the arena (Suppl. Table S1), as has been found in animal studies in the MWM (female meadow voles, Galea et al. 2002). We only found men to be less idle, corroborating the many reports that men simply tend to spatially cover more ground in mazes (Mueller et al. 2008).

Our women on E2V were additionally slower in spatial memory tasks (town: pointing RT; arena: navigation duration during drop-offs) and less efficient over time (arena: LISAS; Fig. 5C). This is similar to the report that intact female rats in the endogenously high-E2 estrous phase took longer to find the target location in MWM than during the low-E2 (but high-P4) diestrus phase or when compared to ovariectomized rats (r.LE: Frye 1995). In human studies, however, there have been reports where women during the high-E2 ovulation phase and during the low-E2 follicular phase performed similarly on a dual-strategy RAM, and performance was best when E2 levels were moderate and decreasing (luteal phase), though strategy learning did not seem to differ across phases (Hussain et al. 2016). We did not find either E2V group to have different navigation distances to target locations than PBO groups (arena: traveled distance, Table 1), although a previous study found that women in the late follicular menstrual phase traversed the shortest path lengths to the target location in a virtual MWM compared to women in the low-E2 and high-P4 mid-luteal phase (Patel et al. 2022). Even if late follicular phase could be considered somewhat comparable to our early follicular phase (high E2 and low P4), E2V administration did not elevate P4 levels in our women (Table 1), and is therefore not comparable to the hormone profile during mid-luteal phase.

However, our observation that E2V groups traveled around the central regions of the arena more than PBO groups (Fig. 3) is an indication of better spatial orientation and/or more confident navigation. E2V groups had reduced path sinuosity in general (Fig. 3 and Suppl. Table S1), suggestive of less certain orientation following the argumentation in the previous section, but also increased direction changes (Suppl. Table S1). These direction changes could be navigators assessing their location to get their bearings. The arena task is notably different from real-world navigation as there are no occluding obstacles or large distances in the arena which might accentuate this effect. However, it should not be forgotten that there are also reports that exogenously elevating E2 levels also lead to worse performance on both striatum- (egocentric) and hippocampus-dependent (allocentric) versions of the RAM, for example, in ovariectomized rats (r.LE: Galea et al. 2001). Not only does dosage level effect different performance (r.LE: Holmes et al. 2002), but regime (r.SD: Gould et al. 1990; r.SD: Miranda et al. 1999; review: Duarte-Guterman et al. 2015). For example, acute administration of low but not high doses of E2 can improve performance on tasks better solved by allocentric studies like the plus maze (Korol and Kolo 2002), T- or Y-maze, RAM and the MWM in female rats compared to ovariectomized controls (Duarte-Guterman et al. 2015). When too high an E2 dose is administered, allocentric spatial performance across numerous tasks is worsened in females (Duarte-Guterman et al. 2015; Frye 1995; Galea et al. 2002; Holmes et al. 2002), consistent with findings that animals with endogenously higher levels of estrogen are slower in spatial learning (e.g., female meadow voles tested on the MWM: Galea et al. 1995). This has led to the suggestion that E2 has a nonlinear effect on behavior: low levels of E2 seem to enhance spatial working memory, high physiological levels of E2 seem to impair, and supraphysiological levels either impair or show no effect (Duarte-Guterman et al. 2015). Our less clear-cut effects of E2 treatment might possibly have been either because we had not elevated E2 enough to elicit a strong effect on our spatial navigation tasks or, given the high E2 levels we had induced (in the high physiological end for women and in the supraphysiological range for men) and the inverted parabolic-shaped E2 dose-to-response in some brain regions but monotonic and linear response in other regions all implicated in spatial navigation (Bayer et al. 2018) and spatial navigation performance (Duarte-Guterman et al. 2015), we might have elevated E2 out of the beneficial range/past the inflection point of optimal performance.

### Reconciling the neuroenhancing role of E2 and the male spatial “advantage”

If E2 has neuroprotecting and neurotrophic actions, including promoting the physiological changes that increase flexibility in spatial navigation and orientation, our results beg the question of why women demonstrate less flexibility in spatial navigation and representation than men. Although animal studies show that E2 can enhance hippocampal-dependent performance in both female and male rodents (Frick 2015; Frick et al. 2018), the mechanisms responsible for these behavior differences might be different, whether due to sex-dependent differences in changes in neuronal morphology (Miranda et al. 1999), in distribution of different ERs (Hyer et al. 2018), or in long-term potentiation (Vierk et al. 2012; Wang et al. 2018) that might play a more critical role than increased spinal density in learning (Smith and McMahon 2005). Although E2 concentration measured in saliva gives a better indication of the concentration in the brain than serum concentration because saliva contains only the bioavailable fraction that can pass the blood-brain barrier in contrast to the protein-bound and unbound E2 present in serum (Bellem et al. 2011), we do not know the actual levels of E2 levels in the brain of our participants. Animal studies have reported that not only is E2 concentration higher in the Hc than in serum, but E2 concentrations are generally higher in males than in females (Ooishi et al. 2012).

Elevated E2 appeared to have a much less of an effect on the spatial behavior in men (Table 1). This is perhaps related to the greater E2-dependent structural and molecular changes observed in females than males (review: Hyer et al. 2018), perhaps partially due to the organizational sex differences in ER distribution and in the mechanisms underlying responses to E2. For example, acute E2 increases induce both the pre- and post-synaptic potentiation that are responsible for the long-term potentiation (LTP) of excitatory synapses in the Hc thought to be responsible for learning (Smith and McMahon 2005) in both males and females, but through different molecular mechanisms. Whereas post-synaptic potentiation is driven by ER$\beta$ in males, it is driven by the membrane-bound G protein-coupled ER 1 (GPER1) in females (r.SD: Oberlander and Woolley 2016). Though GPER1 distribution between the sexes is similar, GPER1-mediated signaling appears to fluctuate in parallel with natural fluctuations in E2 levels in female mice (m.B6: Waters et al. 2015). The immunoreactivity of ER$\alpha$, which is found in higher densities in females, also fluctuates with endogenous E2 levels (r.SD: Oberlander and Woolley 2016). Although it is ER$\beta$ that is found to drive presynaptic potentiation in females instead, while ER$\alpha$ drives it in males, ER$\alpha$ is responsible for activating kinases for LTP only in females (r.SD, m.MOER, m.NOER, m.B6N-WT: Wang et al. 2018). Although ex-vivo studies show that exogenously increasing E2 within physiological ranges in adult male hippocampal slices leads to physiological effects suggesting E2 induces LTP (Kramár et al. 2013), blocking E2 production in vivo does not block LTP generation in male mice as it does in female mice (m.B6 and r.W: Vierk et al. 2012). Additionally, E2-induced potentiation requires a higher threshold in female hippocampal neurons than in males (r.SD, m.MOER, m.NOER, m.B6N-WT: Wang et al. 2018).

These molecular differences can partly explain the differences between the sexes in the magnitude of estrogen-dependent neuronal plasticity with observable changes in cognition. Acute administration of E2 (r.SD: Korol and Kolo 2002) or ER modulators (r.SD: Velázquez-Zamora et al. 2012) to ovariectomized rats can improve allocentric spatial navigation in T- or Y-mazes, accompanied by increased density in dendritic spines (Velázquez-Zamora et al. 2012). Females generally have double the spine density in the Hc of males, which is not observed after ovariectomy (r.SD: Gould et al. 1990). Estrogens can increase dendritic spines in the male Hc but only via rapid actions whereas androgens increase spines both rapidly and over long term. In contrast, E2 increases dendritic spine and synapse density in both rapid and slower actions in the female Hc (Sheppard et al. 2019).

Neurogenesis is found in animal studies with associative learning — even after just a single trial of learning in the MWM, which can moreover be suppressed by ovariectomy (Fx-r.SD: Beltrán-Campos et al. 2011). However, neurogenesis alone does not seem necessary nor sufficient for learning: blocking neurogenesis can impair learning or even not affect learning performance (review: Leuner et al. 2006), as was the case in the hippocampal dental gyrus in male rats in the MWM (r.SD: Shors et al. 2002).

#### TST vs. E2

Although we only manipulated E2 levels exogenously, we should also consider the confounds that TST was unavoidably naturally higher in men than women on Day 1, that our E2V administration reduced TST levels in the male treatment group (M.E2V) likely through negative feedback on the hypothalamo-pituitary-gonadal (HPG) axis (Rune et al. 2023), and that increasing E2 levels increases potential competition for E2 and TST binding on ERs (Stoffel-Wagner et al. 1999). As with E2 dose-response relationships, TST has an inverted parabolic dose-response relationship for some behavior, such as spatial navigation behavior (Mx-r.SD in an MWM: Spritzer et al. 2011) and memory (Gouchie and Kimura 1991), though the relationship is likewise actually more complex (Spritzer et al. 2021). Also like E2, increased (decreased) TST levels are associated with better (worse) cognitive performance that vary with sex, including spatial tasks and memory performance (Burkitt et al. 2007; Kight and McCarthy 2020), though acute, exogenous increases of TST in women by 25-fold in serum reportedly leads to limited enhancement of spatial navigation through a virtual town (Pintzka et al. 2016). This complex response function might partially account for great enough variance resulting in some studies finding endogenous TST levels to correlate with performance in a virtual version of the MWM (vWM) in women but not in men (Burkitt et al. 2007), while others to find this to be the case for men but not women (albeit for a route-learning task using different metrics: Choi and Silverman 2002). Likewise, the many sources of confounding factors could also be why high endogenous TST does not always lead to enhanced spatial navigation flexibility as was found in a study on boys with a rare disorder of androgen excess (familial male precocious puberty) that found no performance differences on a virtual MWM between patients and healthy boys (Mueller et al. 2011) — though we should caution in synthesizing across all these studies as the effects of chronically elevated TST, and, more precisely here, organizational effects of TST, might not be comparable to that of acutely elevated concentrations (Spritzer et al. 2011).

As mentioned in the Introduction, E2-dependent pathways might be responsible for some TST effects on cognition (Williams et al. 1990; Williams and Meck 1993; Wu et al. 2009), including spatial navigation such as route learning (Choi and Silverman 2002), but androgen-dependent pathways also appear to play critical roles in certain activational aspects of spatial cognition (reviews: Frick 2015; Frick et al. 2015; Gibbs 2005; MacLusky et al. 2006). Brain regions consistently implicated in learning and memory, like the Hc, also have a high density of androgen receptors (Moghadami et al. 2016) and are hence sensitive to androgen-mediated effects. Like E2, TST can increase synapse density but is not redundant to E2: it is TST but not E2 that can effectively restore spine synapse density in the posterior Hc (specifically subfield CA1 in x-r.SD: MacLusky et al. 2006; also see Leranth et al. 2003), which expresses many androgen receptors (Kight and McCarthy 2020). Numerous studies have shown that TST increases neurogenesis and survival in new neurons in the Hc and also have neuroprotective properties like estrogen (review: Spritzer et al. 2021). TST enhancement of learning and memory may be partly due to the action of TST metabolites on ER$\beta$ in the dorsal (posterior) Hc (Edinger and Frye 2007). As discussed above, ER$\beta$ is responsible for driving presynaptic potentiation in females but not in males. If circulating TST plays a critical role in spatial navigation cognition, this difference in immunoreactivity and roles of the different ERs between the sexes might partially account for the slower learning and slower performance in women with low TST levels (mean saliva [TST] 45 pg/mL) compared to women with high TST levels (148 pg/mL) who performed comparably to men whether with low (112 pg/mL) or high TST (255 pg/mL) when completing an open arena task equivalent to the MWM (Burkitt et al. 2007). We cannot fully assess the spatial-flexibility enhancing effects of circulating TST in our study, given that TST levels were constant over E2V treatment except for the male groups. If TST were the only critical hormone in spatial navigation cognition and we were to use the Burkitt study as reference (and assuming that the different variables they tested for allocentric strategies are comparable to our proxies of spatial navigation flexibility), we would also expect no enhanced allocentric approaches in any group except possibly between our male groups where E2V treatment lowered TST levels — although our pre- and post-treatment groups had TST levels that both fall in the low-TST male group in the study by Burkitt et al. . We did find a difference between our M.PBO and M.E2V groups (higher vs. lower TST levels, respectively), with M.PBO/high-TST navigating with more direction changes (Table 1, # x-/y-flips). Therefore, if TST enhances allocentric/flexible navigation strategies, #x-/y-flips, which was higher in M.PBO who had higher TST levels, might very plausibly indicate navigation flexibility, therefore supporting our interpretations of it.

Additionally, if TST were the only critical hormone, we should be less likely to find as many navigation differences between our F.E2V and F.PBO groups, who had comparable TST levels but differed in E2 levels, as we have. Therefore, although we have focused on the role of E2 in enhancing spatial navigation flexibility in both our experimental design and discussion and can only make limited comments about the role of TST due to our experimental design, our results attest to the impact of the fluctuations in circulating levels of both E2 and TST and the difficulty in only considering the impact of one irrespective of the other.

### Limitations and conclusions

One of the major limitations of our study, which is common to all human studies, is that we could not completely separate the effects of E2 from TST. The lowered TST in men administered E2V (Fig. 1A) might have mitigated E2 impact on behavior and limits the conclusions we can make of the direct impact of E2, as TST might enhance spatial performance when bioavailability of both TST and E2 are high (Taylor et al. 2017). In women, E2V administration did not affect progesterone levels significantly, however, suggesting that our results are less likely to be directly influenced by varying P4 levels, which have been argued to possibly be the main culprit in the contradictory results in studies of E2 on spatial cognition (Chesler and Juraska 2000), perhaps in mitigating any E2-related enhancements (Hussain et al. 2016).

Another major limitation of our study, also common to all healthy human studies, is our inability to completely separate organizational from activational effects of hormones. Despite our attempt to isolate activational from organizational effects of E2, the critical role of experience (Burigat and Chittaro 2007; Uttal et al. 2013), including that which is gained by culturally imposed gender roles (Hoffman et al. 2011; Trumble et al. 2016; Wood et al. 2021; Vashro and Cashdan 2015) and hence shaping experience, on performance on these tasks should not be underestimated. Even single trial training on the MWM has been found to increase hippocampal dendrite density, accompanied by better performance (Beltrán-Campos et al. 2011). Also to consider is the evolutionary impact of persistent experience that has been suggested to explain, for example, men’s dead-reckoning accuracy with landmarks or consumer items in modern environments like shopping malls (Tlauka et al. 2005), but women’s superior dead-reckoning accuracy which increases with the caloric value of foods in a market (New et al. 2007). Human studies cannot cleanly separate *sex* from *gender* differences, though we did our utmost to tightly design our experimental paradigm within these limitations. Cognizant of this, we have tried to avoid labeling differences as *sex differences* but as *differences between the sexes*.

The suggestions from findings that sex differences are a function of training in both rodents (Bimonte and Denenberg 1999) and humans (Astur et al. 2016; Simpson and Kelly 2012), as well as difficulty (r.PW in RAM: Bimonte and Denenberg 1999) also encourage us to shy away from the categorical/dichotomous view of allocentric vs. egocentric strategies and recast the findings in terms of flexibility. Of note is that castration, and hence eliminating circulating TST, does not eliminate the ability to learn spatial navigation tasks but slowed learning rate (r.SD on RAM and MWM: Spritzer et al. 2011). We would argue that the observation that differences between the sexes appear only when the task becomes difficult (Coluccia and Louse 2004) is also related and fits into our view that sex differences might not necessarily be dichotomous strategy preferences but about flexibility in spatial representations. Although much of our results point to the general interpretation that E2 seems to enhance egocentric and disfavor allocentric spatial navigation, given the equivocal findings across both human and animal studies (Coluccia and Louse 2004; Williams and Meck 1991) and parts of our findings here that do not follow this trend and based on our arguments above, we would argue for going away from the egocentric versus allocentric dichotomy and suggest that spatial cognition could be better characterized as adaptability or flexibility and not be considered as a specialized cognition but based on general, distributed learning (Korol and Wang 2018) — just as the effects of E2 are systemic and is better viewed as multilevel impact from fundamental neuronal structure (Korol and Wang 2018) to the ensemble of neural networks supporting general cognition (Ekstrom et al. 2014).

We had also opted for a translational approach, adopting adaptations of common paradigms used in animal research in order to take advantage of the more controlled paradigms afforded by animal studies and hence the access to testing physiological and cellular mechanisms underlying observed effects. As already briefly discussed, we have glossed over sex, species, and strain differences in spatial navigation tendencies. For example, synapse morphology changes depending on strain in both mice (Wahlsten et al. 2005) and rats (Manahan-Vaughan and Schwegler 2011). Different exploration tendencies and learning curves on common paradigms like the Y-maze, radial mazes, and MWM have been observed (Hok et al. 2016). De novo synthesis of E2 in the Hc and androgen receptor reactivity also vary widely (Hamson et al. 2016). Although we do not directly address this issue, we have tried to be as transparent as possible and conservative in interpreting our results in context of animal studies.

It could be the case that our results were also confounded by some limitations in our methodology. For practical reasons, we were only able to run one of the tasks in the MRI scanner, the arena task, which was the only free navigation task we had. We have synthesized results across all tasks, whether performed inside or outside of the MRI scanner, though performance is known to differ depending on the environment, with reports that participants are slower and commit more decision-making errors in the MRI scanner (van Maanen et al. 2016), possibly because scanner noise negatively affects accuracy in tasks requiring visual attention (Kobald et al. 2016). Different testing environments might also have been compounded by any performance differences on our virtual navigation tasks due to video game experience. Video game players are known to have better navigation skills and more easily create cognitive maps in virtual environments (Murias et al. 2016). We had chosen desktop virtual environments (VE) to approximate real-world navigation. Like in any experiment, design of the VE is crucial and alters behavior observed that might not reflect real-world contexts (Ross et al. 2006). VE additionally lack body-based cues activating the sensorimotor and proprioceptive as well as vestibular systems (Taube et al. 2013) that are arguably critical in real-world navigation and are better approximated by immersive virtual reality (VR) environments. However, there are indications that spatial learning and blood-oxygen level dependent responses in key brain regions implicated in spatial learning do not differ between the physically more restricted VR environment despite the lack of body-based cues and immersive VR (Huffman and Ekstrom 2019), therefore mitigating these concerns somewhat.

Finally, we have been careful to refer to spatial *navigation* differences (see Suppl. Info for a comment on the mental rotation task), that is, focusing specifically on spatial navigation.

Despite these many caveats, physiological sex differences in cognition involved in spatial navigation and memory appear non-negligible (Gaulin 1992), as our results also suggest. In summary, overall, animal studies report that estrogen generally promotes physiological and brain structural changes that should enhance spatial navigation ability, but behavioral effects of circulating estrogen are not clear in either human or animal studies and seem to depend on a complex set of interacting factors such as choice of task, physiological state of the participant, and estrogen concentration. The effects of any differences can also be subtle, as we have shown here for a variety of spatial navigation tasks.

### Supplementary Information

Below is the link to the electronic supplementary material.

Supplementary file 1 (pdf 238 KB)

## Footnotes

1 Unless literature cited is a review or clearly indicated to be human studies, references will be annotated with the species (r: rats, m: mice) and strain (r.SD: Sprague-Dawley/CD strain rats, r.LE: Long-Evans hooded rats, r.W: Wistar rats, r.PW: Purdue-Wistar rats; m.B6J: C57BL/6J mice; WT: wild type) of the animals tested. x-: gonadectomized. Animal studies have demonstrated that exposing male rats to E2 benzoate (EB) or an antiandrogen prenatally can result in hippocampal neurons with morphology and navigational behavior in adulthood that are more similar to female rats than control male rats (r.SD, in the open-field Morris Water Maze, MWM: Isgor and Sengelaub 1998; Similar morphological and behavioral effects in female rats exposed to an androgen were also observed, discussed further in the “TST vs. E2” section). Another study which exposed female rats to EB in the first ten days postnatal similarly found spatial navigation accuracy during adulthood to be comparable to control male rats while neonatally castrated males performed more similarly to control females (r.SD in a radial arm maze, RAM: Williams et al. 1990).
