# Supplementary material for: Effects of estrogen on spatial navigation and memory
Source: Psychopharmacology (Berl). 2024 Feb 26;241(5):1037–63. doi: 10.1007/s00213-024-06539-3 (PMC11031496; doi:10.1007/s00213-024-06539-3)
Supplement: Supplementary file 1 — (pdf 238 KB) [file 213_2024_6539_MOESM1_ESM.pdf]

Supplementary Information

Effects of estrogen on spatial navigation and memory

by G Joue, T Navarro-Schröder, J Achtzehn, S Moffat, N Hennies, J Fuß, C Döller, T Wolbers & T Sommer.

Additional Results

Details of regression models and results are shown in Table S1.

Relation to the classic non-navigation-based spatial task: mental rotation

Other tasks testing general spatial ability are often conflated with spatial navigation. A common such task is the mental rotation task (e.g. of 3-D blocks: Shepard and Metzler 1971; or perspective line drawings of objects: Vandenberg and Kuse 1978). Mental rotation performance has been found to be correlated with faster wayfinding through a maze, virtual (Moffat et al, 1998) or real (Malinowski, 2001), and with fewer errors but only after the maze environment has been learned – but also correlated with tasks involving an egocentric strategy (Moffat et al, 1998). Indeed, mental rotation tasks can be solved with either egocentric or allocentric views (Zacks et al, 2002) and is arguably not a naturalistic task with weak and variable correlation with other spatial navigation tasks.

Mental rotation performance have been reported to fluctuate with E2, though reports are not consistent in women (Noreika et al, 2014; Shirazi et al, 2021; Vashro and Cashdan, 2015) or in men (Peragine et al, 2020). Otherwise, males tend to perform better, though this advantage decreases when more items are tested (Voyer et al, 1995), suggesting that performance differences stem from stress more than ability and that women become more relaxed in test situations over time and hence perform better.

Table S1 Regression model predictions of (back-transformed) spatial navigation metrics reflecting strength of cognitive maps and spatial orientation.

	metric estimates					
predictor	dist/path$^{(O)}$ $e^{log-norm}$	idle time$^{(O)}$ $e^{log-norm}$	y-flips$^{(O)}$ *CMP*	LISAS$^{(O),1}$ *log-log*	sinuosity$^{(O),2}$ $e^{log-norm}$	pt RT$^{(T)}$ *inv.G* (10^{-3})
(Intercept)	2996.0*	9.6*	1.2*	3.6*	0.01*	5.2*
	[2367.1, 3792.0]	[7.7, 12.1]	[1.2, 1.3]	[3.5, 3.7]	[0.01, 0.02]	[3.6, 7.1]
Sex_M	**1.2**	**0.7***	**1.3***	**−0.2***	**1.3***	−0.6
	[1.0, 1.3]	[0.5, 0.9]	[1.3, 1.4]	[−0.3, −0.1]	[1.1, 1.4]	[−3.0, 1.7]
Grp_{E2}	1.1	1.2	**1.2***	−0.02	0.90$^+$	**−1.9**$^+$
	[1.0, 1.2]	[0.9, 1.6]	[1.1, 1.2]	[−0.1, 0.1]	[0.8, 1.0]	[−4.1, 0.2]
Sex_M:Grp_{E2}	0.9	0.9	**0.8***	0.08		1.3
	[0.8, 1.1]	[0.6, 1.2]	[0.8, 0.9]	[−0.1, 0.2]		[−1.6, 4.3]
$^1\log(t_{loc_l})$ or $^2t_{path_p}$				**−0.2***	**1.0***	
				[−0.2, −0.2]	[1.0, 1.0]	
$^1Sex_M$:$\log(t_{loc_l})$ or $^2Sex_M$:t_{path_p}				**−0.02***	**1.0***	
				[−0.04, −0.00]	[1.0, 1.0]	
Grp_{E2}:Sex_M:$\log(t_{loc_l})$				**−0.03***		
				[−0.1, −0.01]		
R^2	$0.0^M/0.5^C$	$0.1^M/0.6^C$		$0.2^M/0.5^C$	$0.01^M/0.13^C$	
Deviance					2.1	
N_{obs}	3732	3732	3732	24031	24148	129
$Ngroup_{Ss}$	129	129		129	129	
$Ngroup_{path}$	36	36				
σ^2	0.5	0.3		0.1	1.02	
τ_{Ss}	0.1	0.3		0.1	0.1	
τ_{path}	0.4	0.1				
$^1\tau_{loc\|Ss}$ or $^2\tau_{path\|Ss}$					0.06 − 0.1	0.00

Task for each metric is shown as a superscript: $^{(O)}$arena, $^{(T)}$town

Metrics: distances to target locations (dist/path), idle time while navigating (idle time), navigation direction changes along the y-axis (y-flips), trade-off between spatial accuracy and time spent navigating (LISAS), sinuosity of navigation paths, and time to complete pointing/dead-reckoning task (pt RT)

The residual variance (σ^2) is reported with between-group variance for random intercept (τ; *loc* location, *Ss* subjects) and random slope ($\tau_{x|y}$ for slope x given y) where applicable.

CMP: Conway-Maxwell Poisson

inv.G: inverse Gaussian with link $1/\mu^2$ for response time for pointing (pt RT)

R^2 variants: ^{M}Marginal/Fixed effects, ^{C}Conditional/Total

Null hypothesis values: * outside the 95% confidence interval or $^+$ at the bounds of the interval.

Colon indicates interaction

References

Malinowski JC (2001) Mental rotation and real-world wayfinding. Percept Mot Skills 92(1):19–30. https://doi.org/10.2466/pms.2001.92.1.19

Moffat SD, Hampson E, Hatzipantelis M (1998) Navigation in a "virtual" maze: Sex differences and correlation with psychometric measures of spatial ability in humans. Evol Hum Behav 19(2):73–87. https://doi.org/10.1016/S1090-5138(97)00104-9

Noreika D, Griškova-Bulanova I, Alaburda A, et al (2014) Progesterone and mental rotation task: Is there any effect? BioMed Res Int 2014. https://doi.org/10.1155/2014/741758

Peragine D, Simeon-Spezzaferro C, Brown A, et al (2020) Sex difference or hormonal difference in mental rotation? the influence of ovarian milieu. Psychoneuroendocrinology 115:104,488. https://doi.org/10.1016/j.psyneuen.2019.104488

Shepard RN, Metzler J (1971) Mental rotation of three-dimensional object. Science 171:701–703

Shirazi TN, Levenberg K, Cunningham H, et al (2021) Relationships between ovarian hormone concentrations and mental rotations performance in naturally-cycling women. Horm Behav 127:104,886. https://doi.org/10.1016/j.yhbeh.2020.104886

Vandenberg SG, Kuse AR (1978) Mental rotations, a group test of three-dimensional spatial visualization. Percept Mot Skills 47(2):599–604. https://doi.org/10.2466/pms.1978.47.2.599

Vashro L, Cashdan E (2015) Spatial cognition, mobility, and reproductive success in northwestern Namibia. Evol Hum Behav 36(2):123–129. https://doi.org/10.1016/j.evolhumbehav.2014.09.009

Voyer D, Voyer S, Bryden MP (1995) Magnitude of sex differences in spatial abilities: A meta-analysis and consideration of critical variables. Psychol Bull 117:250–270. https://doi.org/10.1037/0033-2909.117.2.250

Zacks JM, Ollinger JM, Sheridan MA, et al (2002) A parametric study of mental spatial transformations of bodies. NeuroImage 16(4):857–872. https://doi.org/10.1006/nimg.2002.1129
